# The Passing of the Functional Disorder

**Published:** 1916-02

**Authors:** 


**A Monthly Review of Medicine and Surgery**

EDITOR AND PUBLISHER, DR. A. L. BENEDICT, 228 Summer St., corner of Elmwood Ave., Buffalo, (Address for all communications. Please make personal and telephone calls before 1 P. M.)

**Third, in age, on the western continent. Independent, but supports professional organization. News limited mainly to Western N. Y. and Penn.**

**Subscription $2.00 a year; $3.00 for two years in advance. Changes and errors in address or failure or duplication of delivery, should be reported immediately.**

Yearly Volume 71. FEBRUARY, 1916 No. 7

### The Passing of the Functional Disorder.

Comparatively recent work by Crile on shock, fright, etc., has demonstrated—barring the subsequent presentation or rebutting evidence sufficient to shatter what appears to be the most obvious, direct testimony—that what have heretofore been regarded as purely functional mental phenomena are really organic in their nature, producing visible effects not only in the cerebral cortex but in the cerebellum, medulla and cord. These effects consist in marked changes in the Nissl staining chromophoric portions of the nucleus, apparently directly related to chemic action on the one hand, and to motor discharges on the other.

The changes in the cells can even be measured by differential counts, quite analogous to blood counts. Unfortunately, the method is obviously not applicable clinically nor, as yet, even by the use of successive determinations in the same experimental animal. However, both at autopsy in the human being and still more definitely in the sacrificed animal, inductive evidence of the strongest character, is available for a criterion of subsequent cases and, by controls, the stages of the process can be noted in animals. Nor would we be surprised if operative technic should be perfected so as to allow the stages of the process to be observed in the same experimental animal by the successive removal of minute portions of the brain.

Crile's researches, important as they are in their immediate application, are even more so in their suggestiveness. Thus far, nothing has seemed more purely functional than mental and nervous states due to shock, fatigue, fright, anger, and even toxaemias. At most, we have found an actual basis for them, in vaso-motor phenomena, and perversions of chemistry. Animals have been tortured hour after hour, by the most elaborate methods, with the result that there is no such thing as fatigue of nervous structures—a result contrary to common sense. Now, thanks to Crile's researches, we have a definite basis for the explanation, not only of fatigue but of various other forms of nervous strain, a suggestion as to the correlation of physical, chemic and psychic causes and effects, definite hints as to the relations of such apparently diverse conditions as fright and hyperthyroidism, toxaemia and shock, "nervousness" and diabetes.

It has long been known that nervous manifestations which could only superficially be considered functional, may be due to renal alterations. So, too, toxaemic, dropsical, and vaso-motor manifestations which would, otherwise, be attributed to functional changes, although obviously with more definite physical explanation, have also long been charged to the same organic lesions. Within a comparatively few years, analogous manifestations have been traced to organic changes in various ductless glands and, in the same category, may be placed the researches on diabetes. It is true that these researches do not agree on the pancreas as the locus of all cases of what is commonly termed diabetes, but this fact is no more remarkable and no more discouraging than that various other groups of symptoms may be traced to more than one organic basis. Hypochlorhydria is, in many instances, definitely ascribable to one or other of several organic states. Hyperchlorhydria, with due allowance for the impossibility of securing histologic material in most cases, has almost positively been traced to a definite hypertrophy of definite cells. Now, with a fresh group of experiments, locating lesions of various so-called functional disturbances—and among them those which have long been considered most typically functional—in the Purkinje cells, it is not unreasonable to hope that we shall, in the comparatively near future, be able to discard this unsatisfactory word altogether.

